# Reducing the Cost of Spinal Fixation Surgeries to Fit the Budgets of Patients From the Low- and Middle-Income Categories to Ensure Affordable and Effective Outcomes

**DOI:** 10.7759/cureus.80986

**Published:** 2025-03-22

**Authors:** Binoy K Singh, Bineta Singh, Aishik Mukherjee, Biswajit Dey, Sumit Kumar, Tamajyoti Ghosh

**Affiliations:** 1 Neurosurgery, All India Institute of Medical Sciences, Raipur, Raipur, IND; 2 Pharmacology, Jodhpur Institute of Engineering and Technology Medical College and Hospital, Jodhpur, IND; 3 Neurosurgery, North Eastern Indira Gandhi Regional Institute of Health and Medical Sciences, Shillong, IND; 4 Pathology, North Eastern Indira Gandhi Regional Institute of Health and Medical Sciences, Shillong, IND; 5 Radiation Oncology, North Eastern Indira Gandhi Regional Institute of Health and Medical Sciences, Shillong, IND

**Keywords:** asia score, implant cost, out of pocket expenditure, spinal injury, spine fixation surgery

## Abstract

Background and objective

Traumatic spinal cord injury often results in significant disability, requiring timely surgical intervention to reduce long-term consequences. In low- and middle-income countries, the fragmented healthcare system and high out-of-pocket costs limit access to spinal fixation surgeries, especially for those in the lower income groups. This study aimed to identify the major cost drivers of spinal fixation surgeries and explore strategies to reduce them, making them more affordable for patients without compromising outcomes.

Methodology

This study, conducted at a tertiary care hospital, included 120 patients with traumatic spinal injuries. We analyzed costs related to surgery, including laboratory, radiology, pharmacy, and implant expenses, and compared a prospective group with cost-reducing strategies to a retrospective group.

Results

A total of 120 patients (60 in each group) participated in the study. The major cost driver identified was the implants, particularly screws and rods, which constituted 59% of the total cost of surgery. In the prospective group, the use of short-segment fixation and less expensive implant materials (e.g., titanium mesh cages and metallic disc spacers) led to a significant reduction in costs compared to the retrospective group. Though the mean cost of the implants was significantly lower in the prospective group, there was no difference in surgical outcomes between the two groups. Post-operative complications and readmission rates were similar in both groups and outcomes in terms of neurological improvement were also comparable.

Conclusion

Implant costs are the primary driver of expenses in spinal surgeries. Using short-segment fixation and cost-effective implants reduce costs without affecting outcomes, improving access for patients belonging to low- and middle-income categories. Further studies are needed to evaluate the long-term cost-effectiveness of this strategy.

## Introduction

Traumatic spinal cord injury is a devastating event and can result in significant disability. Timely management, which usually requires fixation, helps in early ambulation and reduces disability [1]. However, the fragmented health delivery structure in India, ossified over the decades, has been slow to keep up with the requirements of the country's massive population comprised chiefly of middle- and low-income groups, hindering the achievement of universal healthcare [2].

The global incidence of traumatic spinal injury is approximately 10.5 cases per 100,000 persons. According to a systematic review and meta-analysis (2022), the incidence of traumatic spinal injuries is more prevalent in low- and middle-income countries (13.69 per 100,000 persons) compared to high-income ones (8.72 per 100,000 persons) [3]. Spinal fusion accounted for the largest aggregate, or 7.1% of costs for hospital stays for any inpatient procedure in the United States [4].

The cost factors in instrumented spinal surgery are varied and include laboratory costs, radiology costs, pharmacy costs, implant costs, readmissions, facility-based costs, surgeon-driven preferences, and patient comorbidities [5]. Each major cost variable represents an opportunity for potential cost reduction, thereby reducing overall costs. The results of spinal surgery are often uncertain. If a patient with a medium or low income has to bear considerable out-of-pocket (OOP) expenses, he/she usually opts out of such surgery. These patients lose the opportunity for early mobilization and recovery and become a burden on the family. With 21.9% of India’s 1.324 billion people living below the poverty line, a high percentage of OOP expenses for treatment can lead to poverty or exacerbate existing poverty [6]. Factors like increased patient cost-sharing, high-deductible health plans, and expensive medications contribute to these expenses. Therefore, the surgeon should try every possible means to reduce the cost of the implant and provide stabilization with acceptable outcomes.

The objectives of this study are to identify the cost drivers of spinal fixation surgery and adopt strategies to reduce the cost of the highest driver, resulting in an overall reduction in cost, thus making it affordable for the patient without changing the outcome.

## Materials and methods

This study was carried out in a single institution, the North Eastern Indira Gandhi Regional Institute of Health and Medical Sciences, a major referral tertiary care center and academic institute of Northeast India that serves the adjoining seven northeastern states. The institute has separate neurosurgery wards, intensive care units (ICU), and on-site imaging facilities for digital X-ray, 3D computerized tomography (CT) scan with angiography and magnetic resonance imaging (MRI). Since this is a tertiary care government center there is no surgeon fee and bed charges are nominal. Hence, these are not included as cost drivers in our study. Patients make payments before imaging and purchase medications unavailable in the hospital. At the time of the study, approximately 35% of the patients had insurance. However, they incurred OOP expenditures for treatment and hospital costs. Since most patients were uninsured, we included all of them in the study to determine the exact costs and enhance the validity of the results. The patients with emergency spinal trauma included in the study were exclusively managed by the neurosurgery department.

The study duration was from December 2020 to November 2021 with 90 days post-study follow-up for the retrospective group and from November 2021 to October 2022 with a similar follow-up period for the prospective group. Sixty patients were included in each group. Participants with an annual salary of Indian rupees (INR) 1.5-5 lakhs were categorized as low- and middle-income patients [7]. Data collected include age, gender, mode of injury, days from injury to admission, location of injury (cervical/dorsal/lumbar), the severity of the injury as per the American Spinal Injury Association (ASIA) scale [8], type of surgery, number of levels stabilized, implants used, hospital stay, complications, and mortality. Both insured and uninsured patients were included in the study.

The surgical methods, the number of implants, and their variety and types were documented. The hospital utilized implants purchased through a tender using rotating funds, as long as they were available. The procedures conducted included spinal decompression and fixation through the following techniques: anterior cervical discectomy and fusion (ACDF), posterior cervical laminectomy and fusion with lateral mass screws and rods, anterior cervical corpectomy and fixation, posterior dorsal and lumbar laminectomy and fusion using pedicle screws and rods, and posterior lumbar corpectomy and fusion with pedicle screws. Postoperatively, patients were encouraged to start ambulation immediately using wheelchairs, physiotherapy, and mobilization.

Cost variables included laboratory expenses, imaging costs, X-rays, CT scans, MRIs, medications (including anesthetics), and implant charges. Implant costs encompassed screws, rods, cages, and plates. The mean costs were calculated, and a p-value was determined for both groups by an independent t-test. A p-value of less than 0.05 was considered significant. Post-operative ASIA scores were compared to assess the outcomes. Neurological status was recorded at discharge and again after 90 days.

Statistical analysis was conducted using means or ranges for various variables to identify which factors contributed the most to the increase in the surgical expenses. We compared the cost per patient of the retrospective group with that of the prospective one by examining the cost drivers in both the groups.

## Results

A total of 120 patients met our inclusion criteria. Sixty patients in each group with a similar age group (16-64 yrs) and type of surgery were studied. The mean age was 34.8 years and 86% of the cohort was male (n=52). In each group, the most common mode of injury was road traffic accidents (35%; n=21), followed by injury due to falls (12%; n=7). Nearly 53% (n=32) of patients in each group suffered from other types of injuries. The most common site of injury was the dorso-lumbar region, followed by the lumbar, and then the cervical and dorsal spine (Table 1).

**Table 1:** Location of the injury and the ASIA score on admission ASIA: American Spinal Injury Association

	Retrospective (n=60)	Prospective (n=60)
Location		
Cervical	18	20
Cervico-dorsal	1	2
Dorsal	4	2
Dorso-lumbar	26	28
Lumbar	11	8
ASIA score on admission		
A	10	9
B	13	12
C	19	23
D	17	14
E	1	2

The mean delay in admission from the date of injury was 10 days for the prospective group and nine days for the retrospective group. Almost 14.2% (n=17) of all the patients had an ASIA score of A on admission (Table 1).

The details of the surgical procedures are provided in Table 2.

**Table 2:** Details of the surgical procedures

Type of operation	Retrospective		Prospective	
	No. of cases	Levels stabilized	No. of cases	Levels stabilized
Anterior cervical discectomy and fusion	5	1	4	1
	1	2	1	2
Anterior cervical corpectomy and fixation	4	1	4	1
			1	2
Posterior cervical laminectomy and fixation	4	2	2	1
	2	3	3	3
	2	4	4	4
Posterior thoracic laminectomy and fixation	4	1	4	1
	1	2	2	2
Posterior dorso-lumbar laminectomy and fixation	20	4	21	3
	6	3	7	2
Lumbar laminectomy and fixation	8	3	7	2
	3	2		

The pharmacy costs, including anesthetics, laboratory, and radiology, constituted around 28% of the total costs. However, there was no significant difference between the two groups (Table 3).

**Table 3:** Laboratory, radiology, and pharmacy costs among the groups CT: Computed tomography; INR: Indian Rupees; MRI: Magnetic resonance imaging; CVP: Central venous pressure

Cost variables	Mean cost in INR (range)		p-value
	Retrospective	Prospective	
Laboratory costs Including blood components	3350 (3053-3558)	3357 (3014-3501)	0.08
Radiology (X-Ray + CT + MRI)	5010 (4120-5902)	5090 (4201-5980)	0.14
Cost of drugs	3552 (3010-4095)	3554 (3032-4076)	0.97
Cost of anesthesia drugs including CVP and arterial line	4553 (4050-5055)	4555 (4075-5036)	0.96

In total, the retrospective group consumed more screws than the prospective group resulting in an increased cost of surgery (Table 4).

**Table 4:** Implant costs among the groups ACDF: Anterior cervical discectomy and fusion; INR: Indian rupees; PEEK: polyetheretherketone

Implants used	Retrospective	Prospective	Mean cost in INR (range)		p-value
			Retrospective	Prospective	
Pedicle screws (titanium)	7-8	4-5	75254 (70184-80120)	65344 (60058-70234)	<0.0001
Cervical lateral mass and rods (titanium)	6-7	4-5	65123 (60156-70043)	55018 (50045-60144)	<0.0001
Rods (titanium)	2	2	11012 (10125-12220)	11015 (10100-12250)	0.97
Anterior cervical plate with corpectomy	Expandable cage 1	Titanium mesh cage 1	Cage: 60523 (50058-70121)	Cage: 22180 (18125-26012)	<0.0001
	Plate 1 with 4 small screws	Plate 1 and 4 screws	20148 (19520-21070)	20156 (19456-21554)	0.92
ACDF spacer	PEEK spacer	Metallic disc spacer (titanium)	23448 (22580-24256)	14553 (13523-15477)	<0.0001

Tables 3, 4 show that the major cost driver was the cost of the implant, which constituted around 59% of the total costs. In the prospective group, a lesser number of screws were used for short-segment fixation and dorsal and lumbar fusion as compared to long-segment posterior fixation conducted in the retrospective group, decreasing the costs in the former group. Similarly, as per the patient's choice, the less-expensive non-expandable titanium mesh cage was used for corpectomy and fixation in the prospective group. Lastly, the prospective group used a titanium disk spacer instead of a polyetheretherketone (PEEK) one, further decreasing expenses.

The mortality rate among the entire cohort was one (0.6%) in each group, due to polytrauma involving blunt chest and abdominal trauma. Seventeen patients (28.3%) improved neurologically in the retrospective group, whereas this number was 13 (21.7%) in the prospective group (Tables 1 and 5).

**Table 5:** Postoperative ASIA scores of patients ASIA: American Spinal Injury Association

Postoperative ASIA score	Retrospective (n=59)	Prospective (n=59)
A	5	7
B	11	10
C	18	21
D	16	13
E	9	8

Four patients in each group (6.7%) had complications due to unintended dural tears and wound infection.

The overall readmission rate was 3.6% at 30 days and 5.4% at 90 days. Revision surgery was required in two patients in the retrospective group and one in the prospective group.

## Discussion

There is a lack of studies on the cost of spinal fixation in low- and middle-income settings, as well as on the cost-effectiveness of spine trauma surgery, even in high-income countries [9,10]. One of the major public health problems affecting low- and middle-income countries is the morbidity and mortality arising out of traumatic spinal injury [11]. The most commonly affected group is the young male patients [12]. In developing countries, traumatic spinal injuries are more common in young adults and males, and motor vehicle crashes and falls are the main etiologies. This is similar to the findings of our study [3]. In a large number of these cases, spinal fixation is indicated, as non-surgical treatment would result in up to a fourfold increase in mortality [12]. The excessive cost of spinal implants often acts as an impediment to surgery in a lot of cases, thereby increasing the burden of the problem [13,14].

Similarly, studies that have evaluated implant costs are limited. Most studies discuss surgeon fees, cost of hospital stay, imaging and emergency room visits, medications, and readmission costs. The study by Reese et al. suggests that facility utilization and supplies/implants are the predominant contributors to the costs (3%) of ACDF [15]. So all possible efforts at lowering costs within these categories should make the most impact on providing cost-effective care, as seen in our study. Modifications of neurosurgery techniques enhance both the affordability and accessibility of the treatment without compromising patient outcomes [15].

In our study, we utilized short-segment fixation in the prospective group to reduce surgery costs. The study by Al Mamun Choudhury et al. indicates that when dealing with fractures at the dorso-lumbar junction, the clinical and radiological outcomes of short-segment fixation are comparable to those of long-segment posterior fixation [16]. This treatment is both a motion segment-saving procedure and cost-effective, as shown in several studies. [17,18]. Short-segment fixation is appropriate even for unstable subaxial cervical spine fractures [19].

We performed anterior cervical decompression with only a titanium spacer which was as clinico-radiologically effective as the PEEK spacer. The type of synthetic graft also did not influence the clinical and radiological outcomes of ACDF with the titanium or PEEK spacer [20,21].

We did not find any difference in the surgical outcomes between the non-expandable and expandable cages in our study. Corpectomy and decompression with instrumented stabilization done in either an expandable or nonexpendable cage is a safe and reliable surgical treatment option for unstable thoracolumbar burst fractures. The expandable cage, however, allowed more correction of the kyphotic deformity [22].

Post-operative complications result in longer hospital stays and significantly higher costs [23]. The readmission rate also increases costs [5]. In the present study, there were complications in four patients in both groups - dural tear in two patients and wound infection in two others. This increased the costs in both groups due to the lumbar drain, wound dressing, antibiotics, and prolonged hospital stay. The readmission rate in the present study was 3.6% post 30 days and 5.6% post 90 days. However, the outcomes in both groups were comparable.

Modifying the surgical methods and implant types in the prospective group helped lower the cost of the surgery without affecting the outcome.

Limitations of the study

This study has several limitations, including being conducted at a single institution, which may limit its generalization to other healthcare settings. It did not account for all potential confounding variables such as socioeconomic factors and comorbidities. The low mortality and complication rates may not reflect the outcomes in higher-risk populations, and the exclusion of long-term costs and post-operative rehabilitation further limits the comprehensive cost analysis. These factors suggest the need for larger, multi-center studies with longer follow-ups to understand the cost-effectiveness and outcomes of spinal trauma surgeries better.

## Conclusions

The costs of spinal surgery in India are much lower compared to the Western countries but are still high for patients from the low- and medium-income categories. The variables that impact the cost of surgery must be determined and strategies should be developed to lower costs for those who cannot afford them, without compromising outcomes. Surgeons can select strategies that involve using cost-effective implant types and reduce the level of fixation for a cost-effective outcome. Proper care should be taken intra-operatively to avoid complications and optimizing patient selection to prevent re-operation and readmissions as they result in increased costs. Limiting the cost of spinal implants can be one of the most effective methods for patients from low- and medium-income groups to undergo essential spinal surgeries.
